# Platelet-Rich Plasma Versus Corticosteroid Injection for Lumbar Spondylosis and Sacroiliac Arthropathy: A Systematic Review of Comparative Studies

**DOI:** 10.7759/cureus.14062

**Published:** 2021-03-23

**Authors:** Jeremiah F Ling, Austin E Wininger, Takashi Hirase

**Affiliations:** 1 Orthopedics and Sports Medicine, Houston Methodist Hospital, Houston, USA

**Keywords:** platelet-rich plasma, corticosteroid, lumbar spondylosis, sacroiliac arthropathy, injection, pain management

## Abstract

This systematic review compares clinical outcomes between platelet-rich plasma (PRP) and corticosteroid injections for the treatment of lumbar spondylosis and sacroiliac arthropathy. A systematic review was registered with the International Prospective Register of Systematic Reviews (PROSPERO) and performed according to Preferred Reporting Items for Systematic Reviews and Meta-Analyses (PRISMA) guidelines using the Pubmed, SCOPUS, and Ovid MEDLINE databases. All level I-III evidence comparative studies published in the English language investigating the clinical outcomes between PRP and corticosteroid injections for the treatment of lumbar spondylosis and sacroiliac arthropathy were included. Five studies (242 patients, 114 PRP, 128 corticosteroid) were analyzed. One randomized study was level I evidence, two randomized studies were level II, and two non-randomized studies were level III. Final follow-up ranged from six weeks to six months. Four studies found that both PRP and corticosteroid treatment led to a statistically significant reduction in the visual analog scale (VAS). One found that only the PRP group led to a statistically significant reduction in VAS. Three studies found more significant improvements in one or more clinical outcome scores among PRP patients as compared with corticosteroid patients at the three- to six-month follow-up. Two studies found no difference in outcome score improvements between the two groups at six- to 12-week follow-up. There were no reports of major complications. There were no significant differences in minor complication rates between the two groups. In conclusion, both PRP and corticosteroid injections are safe and effective options for the treatment of lumbar spondylosis and sacroiliac arthropathy. There is some evidence that PRP injection is a more effective option at long-term follow-up compared with corticosteroid injection. Further randomized controlled trials with longer-term follow-up are necessary to compare its long-term efficacy.

## Introduction and background

One of the leading causes of disability in the United States is chronic low back pain (CLBP), with nearly an 80% lifetime prevalence [1-3]. Low back pain has an enormous economic cost in the United States, as this condition is associated with treatment costs of around $200 billion and 149 million days of work missed per year [4-5]. By definition, CLBP is pain that persists for more than three months and is associated with an increased need for opioid use, spinal injections, and spine surgery in the last two decades [6-8]. Clinical pain syndromes related to CLBP include osteoarthritis, disk degeneration, prior spinal trauma, and lumbar radiculopathy, which are often categorized within the broader term of lumbar spondylosis [9-10]. Sacroiliac arthropathy is closely related to lumbar spondylosis, is another major source of CLBP, and refers to conditions associated with the sacroiliac joint [11]. Currently, non-operative treatment with exercise and the administration of non-steroidal anti-inflammatory drugs (NSAIDs) are often first-line treatments for CLBP and lumbar spondylosis [9-12]. However, clinical practice guidelines report inconsistent exercise recommendations for CLBP, with limited evidence regarding clinically significant benefits with this treatment [12-14]. Furthermore, there are gastrointestinal, cardiovascular, and renal adverse effects that are often associated with chronic NSAIDs use [12-13]. With regard to sacroiliac (SI) arthropathy, corticosteroid injections into the SI joints are often used as an effective treatment option but have been shown to provide only acute relief with limited long-term benefit [11]. Epidural corticosteroid injections have also been recommended as a treatment for CLBP and lumbar spondylosis, with greater acute relief than NSAIDs, a lower side-effect profile, and less systemic effects [15-18]. However, the efficacy of epidural corticosteroid injections for CLBP and lumbar spondylosis is limited and provides solely acute relief and little long-term benefit [15-18].

It is important to understand the biology and underpinnings of lumbar spondylosis in order to avoid only temporal, symptomatic relief, i.e., with NSAIDs and steroid injections. Various factors, including apoptosis, collagen abnormalities, ingrowth of the vasculature, and upregulation of matrix collagenases, have been associated with the disc degeneration seen in lumbar spondylosis [9,19]. In both lumbar spondylosis and sacroiliac arthropathy, the proposed etiology of these conditions involves a cascade of degenerative damage that is associated with the accumulation of microtraumas and changes in forces on joints [9-11]. Changes in the microenvironment, including osteophyte formation as well as matrix collagenase activation, have been attributed to the development of disc degeneration, nerve irritation, and the disruption of cartilage and bone [9-11].

Platelet-rich plasma (PRP) is autologous blood that contains platelet concentrations above normal physiological levels, and the injectable solution is obtained by using centrifugation to separate solid and liquid blood components [20-21]. PRP is believed to stimulate regeneration through the release of growth factors and proteins that may be involved in repairing the matrices of degenerative discs [21]. There are a few systematic reviews and meta-analyses in the literature assessing the efficacy of PRP for treatment in lumbar spondylosis and sacroiliac arthropathy [22-23]. However, these studies are limited, as they lack high-quality randomized controlled trials (RCTs) or the analysis is non-comparative in nature. The most recent systematic review by Xuan et al. assessed prospective randomized controlled trials comparing PRP to control treatments, but this study was limited in that it reviewed only three RCTs in the analysis [22]. Another recent review by Burnham et al. also assessed PRP for sacroiliac arthropathy, utilizing three RCTs in their review, however, they were also limited by the number of available studies [24]. To our knowledge, there is not yet a comprehensive comparative review of PRP injections to corticosteroid injections for the treatment of lumbar spondylosis and sacroiliac arthropathy. The purpose of this study was to develop a comprehensive systematic review of comparative studies in the current literature that compares PRP injections to corticosteroid injections as a therapy for lumbar spondylosis and sacroiliac arthropathy. The authors hypothesized that PRP injection is more effective than corticosteroid injection for the treatment of lumbar spondylosis and sacroiliac arthropathy with a similar safety profile.

## Review

Methods

This systematic review was registered with the International Prospective Register of Systematic Reviews (PROSPERO) on 1/11/2020 (Registration ID: CRD42021230383). The authors were unable to identify any similar prior systematic reviews or meta-analyses within the PROSPERO. The search was conducted and reported using the protocol described in the Preferred Reporting Items for Systematic Reviews and Meta-analyses (PRISMA) guidelines [25].

Two authors conducted separate searches using the following medical databases on January 12, 2020: PubMed (1966-present), SCOPUS (1966-present), and Ovid MEDLINE (1946-present). To ensure a stringent search strategy of relevant literature, keywords including “platelet-rich plasma,” “corticosteroid,” “sacroiliac,” and “spine” were combined with Boolean operators to develop a search protocol. A hand search of the included references was also performed to further minimize the unintentional exclusion of relevant studies.

All Level I, II, and III evidence (as defined by the Oxford Centre for Evidence-Based Medicine (CEBM)) therapeutic studies published in the English language that investigated clinical outcomes between PRP and corticosteroid injections for spinal pathologies were included [26].

**Figure 1:**
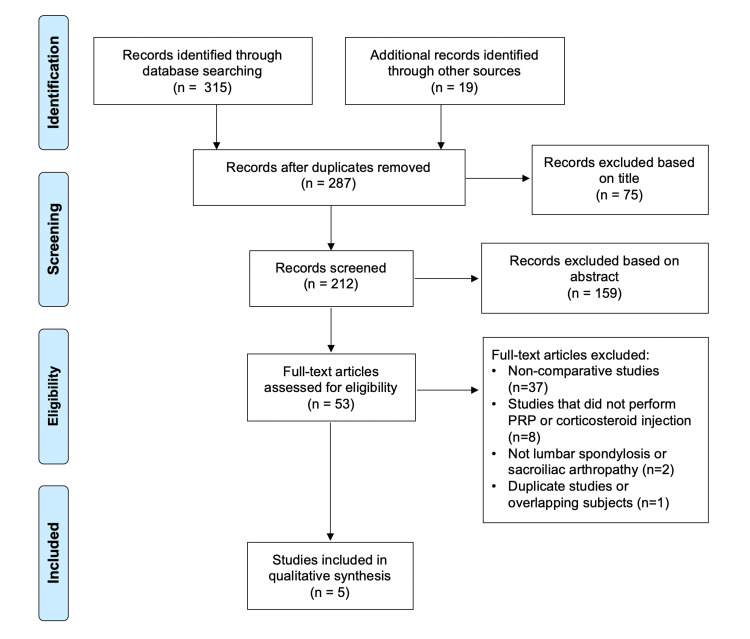
Preferred Reporting Items for Systematic Review and Meta-Analysis (PRISMA) flowchart showing the application of selection criteria to the studies identified with the search strategy

Studies were excluded if they were studies of non-spinal pathology, non-comparative studies (i.e. only one treatment method studied), cadaveric studies, basic science studies, animal studies, diagnostic studies, economic studies, prognostic studies, letters to editors, review articles, editorials, and surveys. In the situation of duplicate studies from the same author(s) and/or institution(s) reporting on the same or overlapping subjects, only one study was retained: the highest level of evidence, the largest number of subjects, the longest follow-up, and the most pertinent primary outcome score (or relevant secondary). The others were excluded (Figure 1).

Two authors independently reviewed all studies using the previously recommended methodology [27]. If two or more separate studies utilized the same patient population, the one with the longer follow-up, higher level of evidence, greater number of patients, and/or greater clarity of methods and results was included. The level of evidence (CEBM), study design, and the methodological quality of each study was graded using the Modified Coleman Methodology Score (MCMS) [26,28]. For all included studies, the overall Strength-of-Recommendation Taxonomy (SORT) score and the Grading of Recommendations Assessment, Development and Evaluation (GRADE) score were calculated [29-30]. Patient demographics (age, gender, diagnosis), adverse events (major and minor), patient-reported outcome scores, and the study authors’ overall conclusion were extracted from each study. For extracting data from digital plots, WebPlotDigitizer version 4.4 (Ankit Rohatgi, Pacifica, CA, USA, https://automeris.io/WebPlotDigitizer) was utilized to best estimate the reported data using prior described methods [31-32]. If the included studies were too heterogeneous, with heterogeneity in study participants, interventions, and/or outcomes, a meta-analysis would not be performed and a systematic review with best-evidence synthesis would be chosen as the synthetic review type [33].

Data analysis was performed using the Statistical Package for the Social Sciences (SPSS) statistical software (Version 25.0; IBM Corp., Armonk, NY). A two-tailed student t-test was used to analyze continuous data and the chi-square test was used to analyze categorical data. A p-value of ≤0.05 was considered statistically significant.

Two authors used the Revised Cochrane Risk-of-Bias Tool for Randomized Trials (RoB 2) and the Risk of Bias in Non-Randomized Studies of Interventions (ROBINS-I) assessment tool to perform a risk-of-bias assessment of each included study [34-35].

Results

Three-hundred and thirty-four studies were identified during the preliminary search, with 47 found to be duplicates. Of the remaining 287 studies, five met all inclusion and exclusion criteria (Figure 1).

One randomized study was level I evidence, two randomized studies were level II, and two non-randomized studies were level III. According to MCMS, two studies were rated as good (scores between 70 and 84) and three studies were rated as fair (scores between 55 and 69) [21]. The overall SORT score was A and the GRADE score was B [21-22]. According to RoB2 and ROBINS-I, the overall risk of bias was low for three studies and moderate for two studies (Table 1). These five studies contained: 242 patients either treated with (i) PRP injection (114 patients) or (ii) corticosteroid injection (128 patients) [36-40]. Three studies included patients with lumbar spondylosis [36,38,40], and two studies included patients with sacroiliac arthropathy (Table 1) [37,39].

**Table 1:** Study demographics included in the analysis Rob2 = Revised Cochrane Risk-of Bias Tool for Randomized Trials; ROBINS-I = Risk of Bias in Non-Randomized Studies of Interventions; MCMS = Modified Coleman Methodology Score; BMI = body mass index; SD = standard deviation; MRI = magnetic resonance imaging; CT = computed tomography; XR = x-ray; PRP = platelet-rich plasma; NR = not recorded; N/A = not applicable

Study	Wu et al. 2017 [36]		Singla et al. 2017 [37]		Mohamed et al. 2018 [39]		Lopez et al. 2020 [38]		Bise et al. 2020 [40]	
Type of Study	Prospective randomized non-blinded controlled		Prospective randomized single-blinded controlled		Prospective non-randomized comparative cohort		Prospective randomized double-blinded controlled		Prospective non-randomized comparative cohort	
Level of Evidence	II		II		III		I		III	
Countries	China		India		Egypt		Spain		France	
Rob2 or ROBINS-I Risk of Bias	Low risk		Low risk		Moderate risk		Low risk		Moderate risk	
MCMS	68 (Fair)		80 (Good)		62 (Fair)		82 (Good)		66 (Fair)	
Type of Spinal Pathology Studied	Lumbar facet arthropathy		Sacroiliac joint arthropathy		Sacroiliac joint arthropathy		Degenerative lumbar spondylosis		Degenerative lumbar radiculopathy	
Method of Diagnosis	Diagnostic intra-articular block		Imaging (XR, MRI or nuclear scan) + 3 or more positive provocative tests		One or more positive provocative tests		MRI or neurophysiological studies		MRI and physical exam findings	
Injection location	Lumbar facet joint(s)	Lumbar facet joint(s)	Sacroiliac joint	Sacroiliac joint	Sacroiliac joint	Sacroiliac joint	Epidural	Epidural	Epidural	Epidural
Dates of Cohort	NR		NR		2013 - 2015		NR		2017-2018	
Groups	PRP	Corticosteroid	PRP	Corticosteroid	PRP	Corticosteroid	PRP	Corticosteroid	PRP	Corticosteroid
Mixture injected	0.5 mL PRP	1 mL betamethasone (5 mg/mL) + 4 mL lidocaine (0.5%)	3 mL PRP + 0.5 mL CaCl_2_	1.5 mL methyl-prednisolone (40 mg/mL) + 1.5 mL lidocaine (2%)	3 mL PRP + 0.5 mL lidocaine	2 mL methyl-prednisolone + 1 mL lidocaine	16.5 mL PRP + 3.5 mL iohexol contrast	20 mL triamcinolone (3 mg/mL) + 3.5 mL iohexol contrast	2.5 mL PRP	2.5 mL hydro-cortisone acetate (25 mg/mL)
Total injection amount per level (mL)	0.5	0.5	3.5	3.5	3.5	3	20	23.5	2.5	2.5
Image-guidance injection technique	Fluoroscopic	Fluoroscopic	Ultrasound	Ultrasound	Fluoroscopic	Fluoroscopic	Fluoroscopic	Fluoroscopic	CT	CT
No. of Subjects	23	23	20	20	16	30	25	25	30	30
Age, mean ± SD	52.9 ± 7.6	52.8 ± 7.3	25.2 ± 12.9	37.0 ± 10.9	38.0		68.0 ± 13.1	61.0 ± 12.6	59.0 ± 15.0	50.0 ± 16.0
Male, n (%)	10 (43.5)	9 (39.1)	16 (80.0)	16 (80.0)	22 (48.8)		11 (45.8)	10 (40.0)	18 (60)	19 (63)
BMI (kg/m^2^), mean ± SD	22.6 ± 1.4	22.4 ± 1.5	23.7 ± 2.5	22.4 ± 2.1	NR	NR	NR	NR	26.0 ± 4.0	25.0 ± 3.0
Duration of pain (months), mean ± SD	19.4 ± 12.3	16.7 ± 12.0	NR	NR	NR	NR	NR	NR	6.9	5.9
Single level treated, n (%)	5 (21.7)	7 (30.4)	N/A	N/A	N/A	N/A	N/A	N/A	30 (100.0)	30 (100.0)
Multiple levels treated, n (%)	18 (78.3)	16 (69.6)	N/A	N/A	N/A	N/A	N/A	N/A	0 (0.0)	0 (0.0)

PRP was obtained in all studies by centrifugation of 30 to 200 mL of autologous blood to perform image-guided injections of 0.5 to 16.5 mL of PRP into the target site (Table 2). Two studies performed epidural injections [38,40], two studies performed sacroiliac joint injections [37,39], and one study performed lumbar facet joint injections [36]. None of the studies recorded the use of post-injection cryotherapy. No study compared leukocyte-poor PRP to leukocyte-rich PRP. However, two studies reported using leukocyte-poor PRP [37,40], and one study reported using leukocyte-rich PRP [38].

**Table 2:** PRP preparation PRP = platelet-rich plasma; NR = not recorded

Study	Wu et al. 2017 [36]	Singla et al. 2017 [37]	Mohamed et al. 2018 [39]	Lopez et al. 2020 [38]	Bise et al. 2020 [40]
PRP Spinning Approach	Double	Single	NR	Single	Single
Spin Speed (relative centrifugal force [*g*])	200 and 400	720	NR	1568	620
Duration of Spin (Min)	10 and 10	15	NR	14	15
PRP Activator	NR	CaCl_2_	NR	NR	NR
Platelet Concentration	4 to 5 times peripheral blood	NR	NR	NR	3 times peripheral blood
Platelet Concentration (/µl)	NR	NR	NR	NR	520 x 10^3 ^± 114 x 10^3^
Leukocyte Amount	NR	Leukocyte-poor	NR	Leukocyte-rich	Leukocyte-poor

Five studies containing 112 PRP patients (98.2%) and 125 corticosteroid patients (97.7%) were available at a final follow-up range of six weeks to six months (Table 3). All five studies reported baseline and final visual analog scale (VAS) scores as the primary outcome measures. Four studies found that both PRP and corticosteroid treatment led to a statistically significant reduction in VAS [36-37,39-40]. Lopez et al. found that only the PRP group led to a statistically significant reduction in VAS [38]. Three studies found a higher reduction in VAS among PRP patients as compared with corticosteroid patients at a follow-up of three to six months that was statistically significant [36-38]. Two studies found no difference in VAS reduction between the two groups at a final follow-up of six to 12 weeks [39-40]. Two studies reported baseline and final Oswestry Disability Index (ODI) as secondary outcome measures [36,40]. Both studies found that both groups led to a statistically significant reduction in ODI. Wu et al. reported a higher reduction in ODI among PRP patients as compared to corticosteroid patients at a follow-up of six months (p<0.05) [36]. Bise et al. found no difference in VAS reduction between the two groups at a follow-up of six weeks [40].

**Table 3:** Clinical outcomes PRP = platelet-rich plasma; f/u = follow-up; SD = standard deviation; ODI = Oswestry Disability Index; VAS = visual analogue scale; RMDQ-24 = Roland-Morris Disability Questionnaire; MODQ = Modified Oswestry Disability Questionnaire; IQR = interquartile range; SF-12 = Short Form 12 survey; MCS = mental composite score; PCS = physical composite score SF-36 = Short Form-36 survey; NR = not recorded; N/A = not applicable *p<0.05 versus corticosteroid group

Study	Wu et al. 2017 [36]		Singla et al. 2017 [37]		Mohamed et al. 2018 [39]		Lopez et al. 2020 [38]		Bise et al. 2020 [40]	
Groups	PRP	Corticosteroid	PRP	Corticosteroid	PRP	Corticosteroid	PRP	Corticosteroid	PRP	Corticosteroid
No. of Subjects	23	23	20	20	16	30	25	25	30	30
Final follow-up	6 months	6 months	3 months	3 months	6-12 weeks	6-12 weeks	6 months	6 months	6 weeks	6 weeks
Patient available at final f/u, n (%)	21 (91.3)	20 (87.0)	20 (100.0)	20 (100.0)	16 (100.0)	30 (100.0)	25 (100.0)	25 (100.0)	30 (100.0)	30 (100.0)
ODI at baseline, mean ± SD	60.7 ± 10.8	59.7 ± 10.3	NR	NR	NR	NR	NR	NR	29.8 ± 9.4	29.8 ± 13.0
ODI at final f/u, mean ± SD	29.7 ± 7.7	44.3 ± 7.0	NR	NR	NR	NR	NR	NR	23.0 ± 12	20.0 ± 14.0
ODI reduction, mean	31.0*	15.4	NR	NR	NR	NR	NR	NR	6.8	9.8
≥ 50% ODI reduction, n (%)	NR	NR	NR	NR	NR	NR	NR	NR	7 (23.3)	12 (40.0)
VAS at baseline, mean ± SD	7.1 ± 1.1	6.7 ± 1.1	6.9	6.2	6.7	6.7 ± 1.0	7.5 ± 1.1	7.2 ± 1.0	6.3 ± 2.2	5.2 ± 2.4
VAS at final f/u, mean ± SD	2.9 ± 1.1	5.2 ± 1.5	1.9	4.3	3.5	3.5 ± 0.8	6.1 ± 1.0	7.5 ± 0.6	4.0 ± 2.2	3.4 ± 2.4
VAS reduction, mean	4.2*	1.5	5.0*	1.9	3.2	3.2	1.4*	-0.3	2.3	1.8
≥ 50% VAS reduction, n (%)	17 (81.0)*	4 (20.0)	18 (90.0)	5 (25.0)	NR	NR	NR	NR	11 (36.7)	13 (43.3)
RMDQ-24 at baseline, mean ± SD	17.2 ± 3.1	17.3 ± 2.2	NR	NR	NR	NR	NR	NR	NR	NR
RMDQ-24 at final f/u, mean ± SD	8.2 ± 3.5	13.6 ± 2.9	NR	NR	NR	NR	NR	NR	NR	NR
RMDQ-24 reduction, mean ± SD	9*	3.7	NR	NR	NR	NR	NR	NR	NR	NR
MODQ at baseline, median (IQR)	NR	NR	44 (36-63)	46 (32-50)	NR	NR	NR	NR	NR	NR
MODQ at final f/u, median (IQR)	NR	NR	11 (5-18)*	27 (15-38)	NR	NR	NR	NR	NR	NR
SF-12 PCS at baseline, median (IQR)	NR	NR	30 (24-33)	29 (24-35)	NR	NR	NR	NR	NR	NR
SF-12 PCS at final f/u, median (IQR)	NR	NR	49 (45-55)*	37 (32-44)	NR	NR	NR	NR	NR	NR
SF-12 MCS at baseline, median (IQR)	NR	NR	33 (26-47)	38 (28-46)	NR	NR	NR	NR	NR	NR
SF-12 MCS at final f/u, median (IQR)	NR	NR	55 (54-58)	49 (42-56)	NR	NR	NR	NR	NR	NR
SF-36 PCS at baseline, mean ± SD	NR	NR	NR	NR	NR	NR	140.1 ± 75.1	141.1 ± 70.2	NR	NR
SF-36 PCS at final f/u, mean ± SD	NR	NR	NR	NR	NR	NR	226.1 ± 61.0*	151.7 ± 84.2	NR	NR

There were no major complications across all five studies (Table 4). Three studies containing 64 PRP patients and 78 corticosteroid patients reported minor complications. The overall minor complication rates, which included injection-related pain, hyperglycemia, and pruritus, were 12.5% and 12.8% for PRP and corticosteroid patients, respectively (p=0.952).

**Table 4:** Adverse events PRP = platelet-rich plasma; Pts = patients; NR = not recorded

Study		Wu et al. 2017 [36]		Singla et al. 2017 [37]		Mohamed et al. 2018 [39]		Lopez et al. 2020 [38]		Bise et al. 2020 [40]	
Groups		PRP	Corticosteroid	PRP	Corticosteroid	PRP	Corticosteroid	PRP	Corticosteroid	PRP	Corticosteroid
No. of Patients		23	23	20	20	16	30	25	25	30	30
Major Complications		0 (0.0)	0 (0.0)	0 (0.0)	0 (0.0)	0 (0.0)	0 (0.0)	0 (0.0)	0 (0.0)	0 (0.0)	0 (0.0)
Pts with 1+ Minor Complications		5 (21.7)	7 (30.4)	NR	NR	2 (12.5)	3 (10.0)	1 (4.0)	0 (0.0)	NR	NR
Minor Complications											
	Injection-related pain	5 (21.7)	7 (30.4)	NR	NR	2 (12.5)	1 (3.3)	0 (0.0)	0 (0.0)	NR	NR
	Hyperglycemia	0 (0.0)	0 (0.0)	NR	NR	0 (0.0)	2 (6.7)	0 (0.0)	0 (0.0)	NR	NR
	Pruritis	0 (0.0)	0 (0.0)	NR	NR	0 (0.0)	0 (0.0)	1 (4.0)	0 (0.0)	NR	NR

Discussion

The authors hypothesized that PRP injection is more effective than corticosteroid injection for the treatment of lumbar spondylosis and sacroiliac arthropathy with a similar safety profile. The first hypothesis was partially disproven as two of the five analyzed studies reported equal effectiveness between PRP and corticosteroid injections in treating pain related to lumbar spondylosis and sacroiliac arthropathy. However, three of the five studies reported more significant improvements in several pain and functional scores among patients receiving PRP as compared to corticosteroid injections at long-term follow-ups. The second hypothesis was confirmed, as both PRP and corticosteroid injections were both noted to be safe with no significant difference in major or minor complication rates between the groups.

To our knowledge, this is the first systematic review in the current literature assessing clinical outcomes between PRP and corticosteroid injections for the treatment of lumbar spondylosis and sacroiliac arthropathy. Although prior systematic reviews and meta-analyses have measured the potency of PRP for pain treatment in lumbar spondylosis and sacroiliac arthropathy, the majority of articles included in these studies were non-comparative in nature [22-24,41]. In the most recent meta-analysis by Xuan et al., prospective randomized controlled studies comparing PRP to other agents (including steroids) were analyzed. However, this study was limited in that it only assessed three studies, one of which only evaluated PRP against a control intra-discal contrast agent [22].

PRP has previously been shown as an effective treatment in tendinopathies, including patellar, Achilles, and humeral epicondyle conditions, as well as degenerative disc disease (DDD) [41]. In a recent study by Hirase et al., PRP was shown to significantly improve VAS scores while also reporting lower complication and reinjection rates for the treatment of DDD, although these findings were limited by the inclusion of non-comparative studies and only assessing VAS as a clinical outcome [41]. Our study provides a more comprehensive comparison, using comparative studies of PRP to corticosteroid injections for the treatment of lumbar spondylosis and sacroiliac arthropathy, and found significant improvements in multiple clinical outcomes, including VAS, ODI, RMDQ-24, and SF-12 in both treatment groups. Another review by Burnham et al. assessed PRP for sacroiliac arthropathy, finding inadequate evidence of PRP as an effective treatment; however, the mean follow-up of their studies was merely three months [24]. Our analysis included studies by Wu et al., Singla et al., and Lopez et al., which all had long-term follow-ups from three to six months. Although these studies initially demonstrated that corticosteroid injections were more effective at short-term follow-up of three to six weeks, PRP injections showed a more significant improvement at long-term follow-up of three to six months [36-38]. The increased effectiveness of PRP seen at a longer follow-up is consistent with the prior systematic review of non-comparative studies by Sanapati et al., with all studies showing significant improvements with PRP at follow-up ranging from six months to 2 years [23].

All five studies reported VAS as an outcome score, and three of these five studies reported a significant improvement in VAS among the PRP groups compared with the corticosteroid group showing significant improvements in VAS scores at long-term follow-up [36-38]. The two studies by Mohamed et al. and Bise et al. with shorter follow-up periods did not report any significant improvement, which supports a time-sensitive efficacy in PRP treatment for lumbar spondylosis and sacroiliac arthropathy [39-40]. However, a major limitation of all five included studies was that they merely reported the mean pre-injection and post-injection outcome scores and did not compare the individual outcome scores with the minimal clinically important difference (MCID). Although statistically significant changes in outcomes scores are reported, they may not reflect clinically significant changes unless individual outcome scores are compared with the MCID [42].

The study by Hagg et al., using a cohort of 289 patients with chronic low back pain, suggests the MCID for VAS to be at least greater than 2.0 [43]. An additional study by Ostelo et al. found the MCID for VAS in chronic low back pain to be 2.5 [44]. In the three studies reporting more significant VAS reductions in PRP groups, all mean changes exceeded 2.5, suggesting that PRP injection provided a clinically relevant benefit in a majority of their patient cohort as compared to the corticosteroid injection group, which the mean VAS improvement did not exceed the lower MCID value reported by Hagg et al. [43]. With respect to ODI, of the two studies reporting ODI, Wu et al. recorded a mean ODI improvement of 31.0 in PRP patients, which exceeded the MCID of 24 established by Hung et al. using a set of 1945 patients [45]. Similarly, Maughan et al. determined an MCID of 5-point change for RMDQ, and in Wu et al., the PRP group reported a mean Roland-Morris Disability Questionnaire (RMDQ) point change of 9.0 as compared to 3.7 in the corticosteroid group, suggesting that a clinically relevant benefit was seen in a majority of the PRP group but not in the corticosteroid group [46].

When considering the treatment of lumbar spondylosis and sacroiliac arthropathy, treatments can be categorized into non-operative and operative. Non-operative treatments for clinical pain syndromes related to CLBP include exercise-based therapy and are often used in conjunction with NSAIDs as part of a treatment strategy; however, these options often may not lead to clinically significant benefits and there are renal, cardiac, and gastrointestinal side effects reported with NSAID usage [9,12,13,47]. Another outpatient treatment option is transcutaneous electrical nerve stimulation (TENS), which involves the delivery of electric stimulation to peripheral nerves to reduce pain. However, TENS has yet to show significant efficacy in the literature and is also ineffective in pain management [9]. Pharmacological agents like opioids and muscle relaxants have also been used in short-term treatments but the neurologic, psychosocial, and gastrointestinal side effects limit their benefit [13-14]. When assessing sacroiliac arthropathy, facet and joint steroid injection is often a preferred treatment modality, however, there is a limited duration of relief provided by steroid injections [11,48]. In the setting of sacroiliac pain resistant to steroid injection, radiofrequency ablation (RFA) is another option, with relief being reported to up to a year, but there is high heterogeneity in the delivery of RFA and what nerves to specifically target [11,48]. Regarding surgical treatment for lumbar spondylosis and sacroiliac arthropathy, the literature is inconclusive as to which patients may be best served by surgical intervention as well as the heterogeneity of different procedures involved in treating numerous pathologies that could lead to pain manifestation [11,48].

This systematic review has several limitations. First, the included studies analyzed a variety of spinal pathologies including sacroiliac arthropathy. However, to our knowledge, this is the first systematic review that comprehensively assesses all comparative studies that analyze PRP versus corticosteroid injection in the spine. Additionally, although we used high-quality comparative studies in this review, only one was of level I evidence. Lastly, it is possible that our stringent search protocol and limiters may have excluded other relevant studies on this topic, including those published in the non-English language.

## Conclusions

Both PRP and corticosteroid injections are safe and effective options for the treatment of lumbar spondylosis and sacroiliac arthropathy. There is some evidence that PRP injection is a more effective option at long-term follow-up as compared with corticosteroid injection. Further randomized controlled trials with longer-term follow-up are necessary to compare its long-term efficacy.
